# ﻿Four complete mitochondrial genomes of the subgenus *Pterelachisus* (Diptera, Tipulidae, *Tipula*) and implications for the higher phylogeny of the family Tipulidae

**DOI:** 10.3897/zookeys.1213.122708

**Published:** 2024-09-27

**Authors:** Yuetian Gao, Wanxin Cai, Yupeng Li, Yan Li, Ding Yang

**Affiliations:** 1 Department of Entomology, College of Plant Protection, China Agricultural University, Beijing 100193, China China Agricultural University Beijing China; 2 Key Laboratory of Economic and Applied Entomology of Liaoning Province, College of Plant Protection, Shenyang Agricultural University, Shenyang, Liaoning 110866, China Shenyang Agricultural University Shenyang China

**Keywords:** Comparative mitogenome, crane fly, phylogenetic analysis, Tipulinae

## Abstract

The complete mitochondrial genomes of Tipula (Pterelachisus) cinereocincta mesacantha Alexander, 1934, T. (P.) legalis Alexander, 1933, T. (P.) varipennis Meigen, 1818, and T. (P.) yasumatsuana Alexander, 1954 are reported, three of them being sequenced for the first time. The mitochondrial genome lengths of the four species are 15,907 bp, 15,625 bp, 15,772 bp, and 15,735 bp, respectively. All genomes exhibit a high AT base composition, with A + T content of 76.7%, 75.0%, 77.8%, and 75.4%, respectively. The newly reported mitogenomes herein show a general similarity in overall structure, gene order, base composition, and nucleotide content to those of the previously studied species within the family Tipulidae. Phylogenetic analyses were conducted to investigate the relationships within Tipulidae, using both Maximum Likelihood and Bayesian Inference approaches. The results show that the four target species of the subgenus T. (Pterelachisus) basically form a monophyletic group within Tipulidae, clustering with species of the *Tipula* subgenera T. (Lunatipula), T. (Vestiplex), and T. (Formotipula); however, the genus *Tipula* is not monophyletic. Moreover, neither the tipulid subfamily Tipulinae nor the family Limoniidae is supported to be a monophyletic group. The monophyly of the family Tipulidae, and the sister relationship between Tipulidae and Cylindrotomidae are reconfirmed. These research findings could contribute to deep insights into the systematic and evolutionary patterns of crane flies.

## ﻿Introduction

The subgenus Pterelachisus Rondani, 1842, comprising approximately 200 species in the world, is one of the most speciose subgenera of the genus *Tipula* Linnaeus, 1758 belonging to Tipulidae, Tipuloidea, Diptera (Oosterbroek 2024). Tipula (Pterelachisus) is widely distributed in the Northern Hemisphere, primarily including the Palaearctic, Nearctic, and Oriental regions (Oosterbroek 2024). The adults of T. (Pterelachisus) are mostly medium- to large-sized, often gray, yellow, or brown, with pruinescence on the body, usually three or four darker stripes present on the prescutum of mesothorax, and more or less conspicuous grayish or brownish markings on the wing. The larvae of T. (Pterelachisus) are believed to be detritivores or herbivores, commonly inhabit forests within humus-rich soil, decaying wood, or beneath moss on dead wood or rock (Brindle 1960; Alexander 2002; Polevoi and Pilipenko 2016; Kramer and Langlois 2019; Gorban and Podeniene 2022; Cai et al. 2023).

Though some revision work on the taxonomy of T. (Pterelachisus) had been done (Savchenko 1964; Alexander 1965; Theowald 1980; Salmela 2009; Pilipenko 2009), it was still difficult to define this subgenus. Due many similarities in both adults and larvae, the boundaries between T. (Pterelachisus) and T. (Vestiplex), T. (Lunatipula), and some other related subgenera of *Tipula*, are frequently confused, which make them loosely termed the “*Vestiplex*-*Lunatipula*” group of subgenera (Gelhaus 1986; 2005). Up to now, only a little phylogenetic work on the relationship within the group had been done based on morphological data (Savchenko 1979; Gelhaus 2005). However, questions about the monophyly and the delimitation of T. (Pterelachisus), as well as the evolutionary relationships between T. (Pterelachisus) and other subgenera remain unsolved.

Mitochondrial genomes typically exhibit a circular structure, with a size ranging from 15 to 18 kb, comprising multiple segments, including 13 protein-coding genes, two ribosomal RNA (rRNA) genes, and 22 transfer RNA (tRNA) genes. The cytochrome c oxidase I (COI) gene has been widely employed as a barcoding marker for species identification (Hebert et al. 2003; Pilipenko et al. 2012; Men et al. 2017; Sharkey et al. 2021). Due to their relatively easy accessibility, stable gene content, relatively conserved gene arrangement, maternal inheritance, and infrequent recombination, mitochondrial genomes have shown significant value in resolving insect taxonomic study and reconstructing phylogenetic relationships over the past few decades (Wilson et al. 1985; Wei and Chen 2011; Cameron 2014). With the advancement of gene sequencing technology, mitochondrial genomes have been frequently used for insect systematic and evolutionary studies, not only at higher taxonomic levels (Timmermans and Vogler 2012; Li et al. 2017; Ding et al. 2019; Zhang et al. 2019c; Lorenz et al. 2021; Lin et al. 2022a, 2022b; Song et al. 2022; Liu et al. 2023; Zhang et al. 2023), but also on inter- or infraspecific groups (Du et al. 2019; 2021). Nevertheless, there has not been much research on crane flies in this area. Beckenbach (2012) was among the pioneers in releasing partial mitochondrial genomes of Tipulidae and using them to delineate phylogenetic relationships within the crane fly infraorder Tipulomorpha. Subsequently, Zhang et al. (2016) and Kang et al. (2023) used complete mitochondrial genomes to explore phylogenetic relationships within Tipulomorpha.

Before this study, only one species of T. (Pterelachisus), T. (P.) varipennis Meigen, 1818, had a partial mitogenome obtained from the whole-genome sequencing data (SRR1469981), which was updated into the NCBI database by Leerhoei in 2020 with the accession number MT410829. In this study, another three species of *Pterelachisus*, including T. (P.) cinereocincta mesacantha Alexander, 1934, T. (P.) legalis Alexander, 1933 and T. (P.) yasumatsuana Alexander, 1954 were sequenced by Next Generation Sequencing (NGS) technology. All complete mitochondrial genomes of the above four species were assembled and annotated. Nucleotide composition, codon use, transfer RNA secondary structure, evolutionary patterns among PCGs (protein-coding genes), and structural elements in the control region were analyzed. Based on these data, plus some previous mitogenomic data of other species, the phylogeny of Tipuloidea was reconstructed using both Bayesian Inference (BI) and Maximum Likelihood analysis (ML).

## ﻿Materials and methods

### ﻿Sampling, DNA extraction, and sequencing

All the specimens of the three species sequenced in this study were collected and identified by authors and the collecting information is summarized in Suppl. material 1. After collection, each specimen was immediately preserved in 95% ethanol, and later stored at -20 °C in the laboratory. The voucher specimen of T. (P.) cinereocincta mesacantha was deposited in the Entomological Museum of Shenyang Agricultural University (**SYAU**), while those of T. (P.) legalis and T. (P.) yasumatsuana were deposited in the Entomological Museum of China Agricultural University (**CAU**). Genomic DNA was extracted from the thoracic tissue of each specimen using the QIAamp DNA Blood Mini Kit (Qiagen, Germany). The DNA concentration was quantified using an Agilent 5400 instrument.

For DNA library preparation, the NEB Next® Ultra™ DNA Library Prep Kit was utilized, and paired-end sequencing was conducted on an Illumina NovaSeq 6000 platform, generating raw data with an insert size of 350 bp and a read length of 150 bp. Approximately 4 Gb of raw sequenced data was obtained. Novogene Biotechnology Company (Beijing, China) conducted the aforementioned processes. The paired raw reads for the whole mitogenome of T. (P.) varipennis was downloaded from NCBI under the accession number SRR11469981.

### ﻿Mitochondrial genomes assembly, annotation, and analysis

The mitochondrial genomes of all species were assembled using IDBA-UD 1.1.3 (Peng et al. 2012), and circularization of resulting linear contigs was verified using the python script in MitoZ 2.3 software (Meng et al. 2019). Circular mitochondrial genomes were then submitted to the MITOS2 web service (Bernt et al. 2013) for annotation. The secondary structure of tRNA was determined using both the MITOS2 web service (Bernt et al. 2013) and the tRNAscan-SE 2.0 (Lowe and Chan 2016) web service. Annotated mitochondrial genomes underwent a comparative analysis with closely related species in Geneious 9.0.2, and manual corrections were applied. Subsequent analyses were conducted after error exclusion.

Gene maps of the mitochondrial genomes sequenced of four T. (Pterelachisus) species were generated using the Proksee web service (Grant et al. 2023). Basal composition and amino acid usage were calculated using PhyloSuite 1.2.3 (Zhang et al. 2020), with AT skew defined as [A – T] / [A + T] and GC skew defined as [G – C] / [G + C] (Perna and Kocher 1995). Ka and Ks values, along with nucleotide diversity (Pi), were obtained using DnaSP 6.12.03 (Rozas et al. 2017). Relative synonymous codon usage (RSCU) data were also acquired through PhyloSuite (Zhang et al. 2020), and Rscript was employed for graphical representation. Repeat segments in the control region (CR) were identified using the Tandem Repeats Finder 4.09 (Benson 1999).

Accurately annotated mitochondrial genomes, along with sequencing data, were deposited in the NCBI database under the BioProject PRJNA1067446.

### ﻿Phylogenetic analysis

A total of 31 complete mitochondrial genomes were used for phylogenetic analysis in this study (Table 1). *Trichocerabimacula* Walker, 1848 and *Paracladuratrichoptera* (Osten Sacken, 1877), members of the family Trichoceridae, were designated as outgroups, serving as the root of the phylogenetic tree. Twenty-nine species within four families of Tipuloidea were contained in the ingroups, which respectively include one species of Pediciidae, ten species within ten genera of Limoniidae, one species of Cylindrotomidae, and 17 species within 12 (sub)genera of Tipulidae. All data preprocessing was carried out using PhyloSuite 1.2.3. Mitochondrial genomes served as the basis for constructing four concatenated datasets: 1) 13PCG, including all three codon positions of 13 PCGs. 2) 13PCG + rRNA, including all three codon positions of 13 PCGs and two Ribosomal RNA genes. 3) 13PCG12, including the first and second codon positions of 13 PCGs. And 4) AA, including all amino acid of 13 PCGs. Prior to concatenation, all data underwent alignment using MAFFT (Katoh and Standley 2013), followed by manual correction in MEGA7 (Kumar et al. 2016) to eliminate gap regions. Model selection for optimal models was performed using PartitionFinder 2.1.1 (Lanfear et al. 2016).

**Table 1.** Taxonomic information, GenBank accession numbers, and references of mitochondrial genomes used in the present study.

Family	Species	GenBank number	Reference
**Outgroup**			
Trichoceridae	*Paracladuratrichoptera* (Osten Sacken, 1877)	NC016173	(Beckenbach 2012)
Trichoceridae	*Trichocerabimacula* Walker, 1848	NC016169	(Beckenbach 2012)
**Ingroup**			
Pediciidae	*Pedicia* sp.	KT970062	(Zhang et al. 2016)
Limoniidae	*Conosiairrorata* (Wiedemann, 1828)	NC057072	(Zhang et al. 2019a)
Limoniidae	*Dicranomyiamodesta* (Meigen, 1818)	MT628560	Direct submission
Limoniidae	*Epiphragmamediale* Mao & Yang, 2009	NC057085	(Zhang et al. 2021)
Limoniidae	*Euphylidoreadispar* (Meigen, 1818)	MT410841	Direct submission
Limoniidae	*Limoniaphragmitidis* (Schrank, 1781)	NC044484	(Ren et al. 2019c)
Limoniidae	*Metalimnobiaquadrinotata* (Meigen, 1818)	MT584154	Direct submission
Limoniidae	*Paradelphomyia* sp.	KT970061	(Zhang et al. 2016)
Limoniidae	*Pseudolimnophilabrunneinota* Alexander, 1933	MN398932	(Ren et al. 2019a)
Limoniidae	*Rhipidiachenwenyoungi* Zhang, Li &Yang, 2012	KT970063	(Zhang et al. 2016)
Limoniidae	*Symplectahybrida* (Meigen, 1804)	NC030519	(Zhang et al. 2016)
Cylindrotomidae	*Cylindrotoma* sp.	KT970060	(Zhang et al. 2016)
Tipuloidea	*Nephrotomaflavescens* (Linnaeus, 1758)	MT628586	Direct submission
Tipuloidea	*Nephrotomaquadrifaria* (Meigen, 1804)	MT872674	Direct submission
Tipuloidea	*Nephrotomatenuipes* (Riedel, 1910)	MN053900	(Ren et al. 2019b)
Tipuloidea	*Nigrotipulanigra* (Linnaeus, 1758)	MT483653	Direct submission
Tipuloidea	*Tanypterahebeiensis* Yang &Yang, 1988	NC053795	(Zhao et al. 2021)
Tipuloidea	Tipula (Acutipula) cockerelliana Alexander, 1925	NC030520	(Zhang et al. 2016)
Tipuloidea	Tipula (Dendrotipula) flavolineata Meigen, 1804	MT410828	Direct submission
Tipuloidea	Tipula (Formotipula) melanomera gracilispina Savchenko, 1960	MK864102	(Zhang et al. 2019b)
Tipuloidea	Tipula (Lunatipula) fascipennis Meigen, 1818	NC050319	Direct submission
Tipuloidea	Tipula (Nippotipula) abdominalis (Say, 1823)	JN861743	(Beckenbach 2012)
Tipuloidea	Tipula (Pterelachisus) legalis Alexander, 1933	PP209204	This study
Tipuloidea	Tipula (Pterelachisus) cinereocincta mesacantha Alexander, 1934	PP209203	This study
Tipuloidea	Tipula (Pterelachisus) varipennis Meigen, 1818	PP209205	This study
Tipuloidea	Tipula (Pterelachisus) yasumatsuana Alexander, 1954	PP209206	This study
Tipuloidea	Tipula (Tipula) paludosa Meigen, 1830	MT483696	Direct submission
Tipuloidea	Tipula (Vestiplex) aestiva Savchenko, 1960	NC063751	(Gao et al. 2023)
Tipuloidea	Tipula (Yamatotipula) nova Walker, 1848	NC057055	(Zhao et al. 2019)

AliGROOVE 1.08 (Kück et al. 2014) was used to offer the possibility to exclude taxa or gene partitions. Phylogenetic analysis for Maximum Likelihood (ML) trees utilized RAxML 8.2.12 (Stamatakis 2014) with specific parameters set as -m GTRGAMMA -x 1234 -p 12345 -# 1000. Bayesian analysis was conducted using MrBayes 2.3 (Ronquist et al. 2012) for 2,000,000 generations with the default settings. The resulting phylogenetic tree was visualized and enhanced for presentation using Figtree 1.4.4 and Adobe Photoshop 2022.

## ﻿Result and discussion

### ﻿Mitogenomic organization and base composition

**Figure 1.**
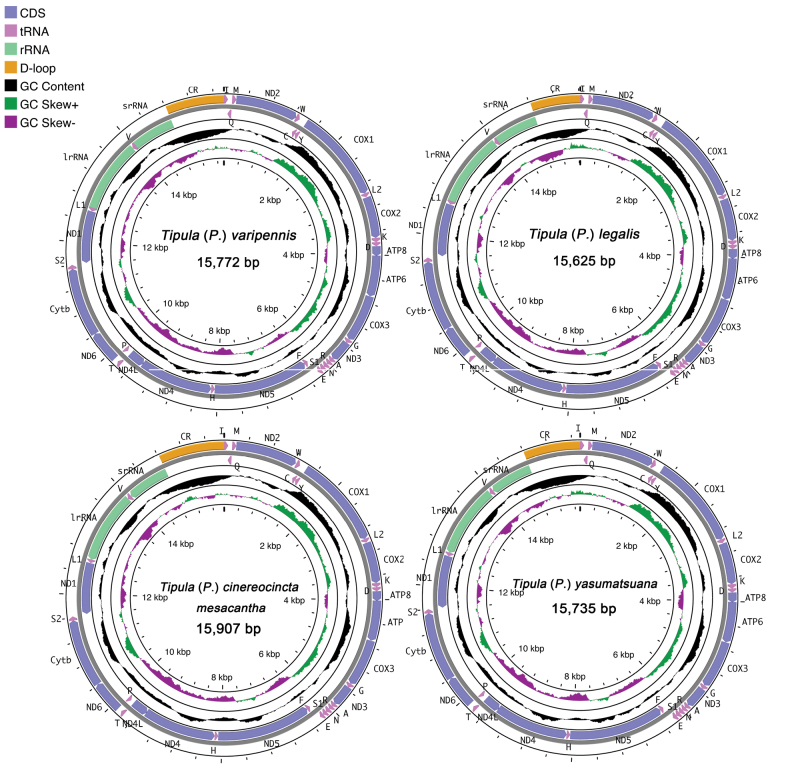
Gene maps of the mitochondrial genomes of the four T. (Pterelachisus) species involved in this study. The transcriptional direction is indicated by arrows.

The complete mitochondrial genomes of all four T. (Pterelachisus) species comprise 13 protein-coding genes, 22 transfer RNA genes, two ribosomal RNA genes, and one non-coding region (A + T-rich control region) (Table 2; Fig. 1). These genes exhibit a ring structure and the tandem arrangement is consistent with previously published mitochondrial whole genome gene arrangements in species of Tipulidae. The total length of the four mitochondrial genomes ranges from 15,000 to 16,000 base pairs. Specifically, T. (P.) cinereocincta mesacantha, T. (P.) legalis, T. (P.) varipennis, and T. (P.) yasumatsuana have lengths of 15,907 bp, 15,625 bp, 15,772 bp, and 15,735 bp, respectively (Table 3). All mitochondrial genomes are notably AT-rich, with A + T base contents for T. (P.) cinereocincta mesacantha, T. (P.) legalis, T. (P.) varipennis, and T. (P.) yasumatsuana at 76.7%, 75.0%, 77.8%, and 75.4%, respectively (Table 3).

**Table 2.** Mitochondrial genome structures of T. (P.) cinereocincta mesacantha Alexander, 1934, T. (P.) legalis Alexander, 1933, T. (P.) varipennis Meigen, 1818, and T. (P.) yasumatsuana Alexander, 1954.

Gene	Strand	Position	Size	Codon	Intergenic nucleotides
***trnI***	H	1-67/1-66/1-67/1-67	67/66/67/67	–	–
***trnQ***	L	65-133/64-132/65-133/65-133	69/69/69/69	–	-3/-3/-3/-3
***trnM***	H	134-202/136-204/137-205/134-202	69/69/69/69	–	0/3/3/0
***nad2***	H	203-1234/205-1236/206-1237/203-1234	1032/1032/1032/1032	ATT-TAA/ATT-TAA/ATT-TAA/ATT-TAA	–
***trnW***	H	1245-1313/1248-1316/1236-1304/1233-1301	69/69/69/69	–	10/11/-2/-2
***trnC***	L	1306-1367/1309-1370/1297-1359/1294-1355	62/62/63/62	–	-8/-8/-8/-8
***trnY***	L	1369-1434/1374-1439/1361-1426/1358-1423	66/66/66/66	–	1/3/1/2
***cox1***	H	1433-2968/1438-2973/1425-2960/1422-2957	1536/1536/1536/1536	TCG-TAA/TCG-TAA/TCG-TAA/TCG-TAA	-2/-2/-2/-2
***trnL2***	H	2969-3032/2974-3037/2961-3024/2958-3021	64/64/64/64	–	–
***cox2***	H	3041-3725/3046-3730/3033-3717/3030-3714	685/685/685/685	ATG-T/ATG-T/ATG-T/ATG-T	8/8/8/8
***trnK***	H	3726-3796/3731-3801/3718-3788/3715-3785	71/71/71/71	–	–
***trnD***	H	3796-3861/3801-3866/3788-3853/3785-3851	66/66/66/67	–	-1/-1/-1/-1
***atp8***	H	3862-4023/3867-4028/3854-4015/3852-4013	162/162/162/162	ATT-TAA/ATT-TAA/ATT-TAA/ATT-TAA	–
***atp6***	H	4017-4694/4022-4699/4009-4686/4007-4684	678/678/678/678	ATG-TAA/ATG-TAA/ATG-TAA/ATG-TAA	-7/-7/-7/-7
***cox3***	H	4697-5485/4702-5490/4689-5477/4687-5475	789/789/789/789	ATG-TAA/ATG-TAA/ATG-TAA/ATG-TAA	2/2/2/2
***trnG***	H	5488-5553/5493-5558/5480-5543/5478-5543	66/66/64/66	–	2/2/2/2
***nad3***	H	5554-5907/5559-5912/5544-5895/5544-5897	354/354/352/354	ATT-TAA/ATT-TAG/ATT-T/ATT-TAA	–
***trnA***	H	5907-5971/5911-5974/5896-5960/5898-5961	65/64/65/64	–	-1/-2/0/0
***trnR***	H	5971-6033/5974-6038/5960-6023/5961-6023	63/65/64/63	–	-1/-1/-1/-1
***trnN***	H	6036-6101/6040-6105/6026-6091/6024-6089	66/66/66/66	–	2/1/2/0
***trnS1***	H	6102-6168/6106-6172/6092-6158/6090-6156	67/67/67/67	–	–
***trnE***	H	6169-6233/6173-6238/6159-6224/6157-6223	65/66/66/67	–	–
***trnF***	L	6266-6331/6266-6331/6257-6322/6251-6316	66/66/66/66	–	32/27/32/27
***nad5***	L	6332-8063/6332-8063/6323-8054/6317-8048	1732/1732/1732/1732	ATG-T/GTG-T/GTG-T/GTG-T	–
***trnH***	L	8064-8129/8064-8129/8055-8120/8049-8114	66/66/66/66	–	–
***nad4***	L	8130-9465/8129-9466/8121-9456/8114-9451	1336/1338/1336/1338	ATG-T/ATG-TAA/ATG-T/ATG-TAA	0/-1/0/-1
***nad4L***	L	9459-9755/9460-9756/9450-9746/9445-9741	297/297/297/297	ATG-TAA/ATG-TAA/ATG-TAA/ATG-TAA	-7/-7/-7/-7
***trnT***	H	9758-9823/9759-9823/9749-9815/9744-9808	66/65/67/65	–	2/2/2/2
***trnP***	L	9824-9889/9824-9887/9816-9880/9809-9873	66/64/65/65	–	–
***nad6***	H	9892-10419/9890-10417/9883-10410/9876-10403	528/528/528/528	ATT-TAA/ATT-TAA/ATC-TAA/ATT-TAA	2/2/2/2
***cytb***	H	10419-11555/10417-11553/10410-11546/10403-11539	1137/1137/1137/1137	ATG-TAG/ATG-TAG/ATG-TAG/ATG-TAG	-1/-1/-1/-1
***trnS2***	H	11554-11621/11552-11619/11545-11612/11538-11605	68/68/68/68	–	-2/-2/-2/-2
***nad1***	L	11638-12579/11636-12577/11629-12570/11622-12563	942/942/942/942	ATA-TAA/ATG-TAA/ATA-TAA/ATG-TAA	16/16/16/16
***trnL1***	L	12584-12647/12582-12645/12575-12638/12568-12631	64/64/64/64	–	4/4/4/4
***rrnL***	L	12648-13966/12646-13966/12639-13961/12632-13954	1319/1321/1323/1323	–	–
***trnV***	L	13967-14038/13967-14038/13962-14033/13955-14026	72/72/72/72	–	–
***rrnS***	L	14039-14821/14039-14820/14034-14815/14027-14809	783/782/782/783	–	–
***control region***		14822-15907/14821-15625/14816-15772/14810-15735	1086/805/957/926	–	–

**Table 3.** Nucleotide composition of mitochondrial genomes of the four T. (Pterelachisus) species.

Species	Regions	Length (bp)	T%	C%	A%	G%	A+T%	AT Skew	GC Skew
T. (P.) cinereocincta mesacantha	Whole genome	15907	38.1	14.2	38.6	9.2	76.7	0.006	-0.214
	PCGs	11205	43.0	12.3	31.6	13.1	74.6	-0.152	0.035
	1^st^ codon position	3735	36.4	11.9	32.2	19.5	68.6	-0.062	0.245
	2^nd^ codon position	3735	46.2	18.8	20.3	14.6	66.5	-0.389	-0.126
	3^rd^ codon position	3735	46.3	6.1	42.4	5.2	88.7	-0.044	-0.073
	tRNAs	1463	37.9	10.0	38.6	13.5	76.5	0.010	0.151
	rRNAs	2102	40.8	6.9	39.4	12.9	80.2	-0.018	0.308
	Control region	1086	46.8	5.8	43.9	3.5	90.7	-0.032	-0.247
T. (P.) legalis	Whole genome	15625	36.7	15.7	38.3	9.3	75.0	0.021	-0.257
	PCGs	11208	41.8	13.6	30.8	13.7	72.6	-0.152	0.004
	1^st^ codon position	3736	35.8	12.7	31.4	20.2	67.2	-0.066	0.229
	2^nd^ codon position	3736	46.0	19.3	20.3	14.5	66.3	-0.387	-0.144
	3^rd^ codon position	3736	43.7	9.0	40.7	6.6	84.4	-0.036	-0.151
	tRNAs	1461	37.9	9.9	39.3	12.9	77.2	0.018	0.135
	rRNAs	2103	40.8	7.0	38.3	13.9	79.1	-0.032	0.327
	Control region	805	47	6.1	44.5	2.5	91.5	-0.027	-0.419
T. (P.) varipennis	Whole genome	15772	39.0	13.1	38.8	9.1	77.8	-0.003	-0.182
	PCGs	11202	43.6	11.5	32.4	12.4	76.0	-0.147	0.040
	1^st^ codon position	3734	36.6	11.8	32.6	19.0	69.2	-0.059	0.237
	2^nd^ codon position	3734	46.3	18.8	20.4	14.5	66.7	-0.390	-0.127
	3^rd^ codon position	3734	48.0	3.9	44.4	3.7	92.4	-0.039	-0.028
	tRNAs	1464	38.5	9.7	38.9	12.8	77.4	0.005	0.139
	rRNAs	2105	40.9	6.9	39.5	12.7	80.4	-0.017	0.296
	Control region	957	47.5	5.3	43.9	3.2	91.4	-0.039	-0.247
T. (P.) yasumatsuana	Whole genome	15735	37.2	15.2	38.2	9.4	75.4	0.013	-0.235
	PCGs	11208	42.5	13.3	30.5	13.8	73.0	-0.164	0.019
	1^st^ codon position	3736	35.7	12.7	31.5	20.2	67.2	-0.063	0.227
	2^nd^ codon position	3736	45.9	19.5	20.2	14.4	66.1	-0.388	-0.149
	3^rd^ codon position	3736	45.9	7.6	39.8	6.7	85.7	-0.071	-0.062
	tRNAs	1463	38.1	10.2	38.7	13.1	76.8	0.008	0.124
	rRNAs	2106	40.9	7.0	38.5	13.5	79.4	-0.030	0.316
	Control region	926	46.7	5.4	44.7	3.2	91.4	-0.022	-0.256

The mitochondrial genomes of the four species share similar, but not identical, intergenic regions and overlaps. The longest intergenic regions, found between *trnE* and *trnF* genes, measure 32 bp, 27 bp, 32 bp, and 27 bp for T. (P.) cinereocincta mesacantha, T. (P.) legalis, T. (P.) varipennis, and T. (P.) yasumatsuana, respectively. The longest overlaps, located between *trnW* and *trnC* genes, are consistent across all species at a length of 8 bp.

### ﻿Protein-coding genes

**Figure 2.**
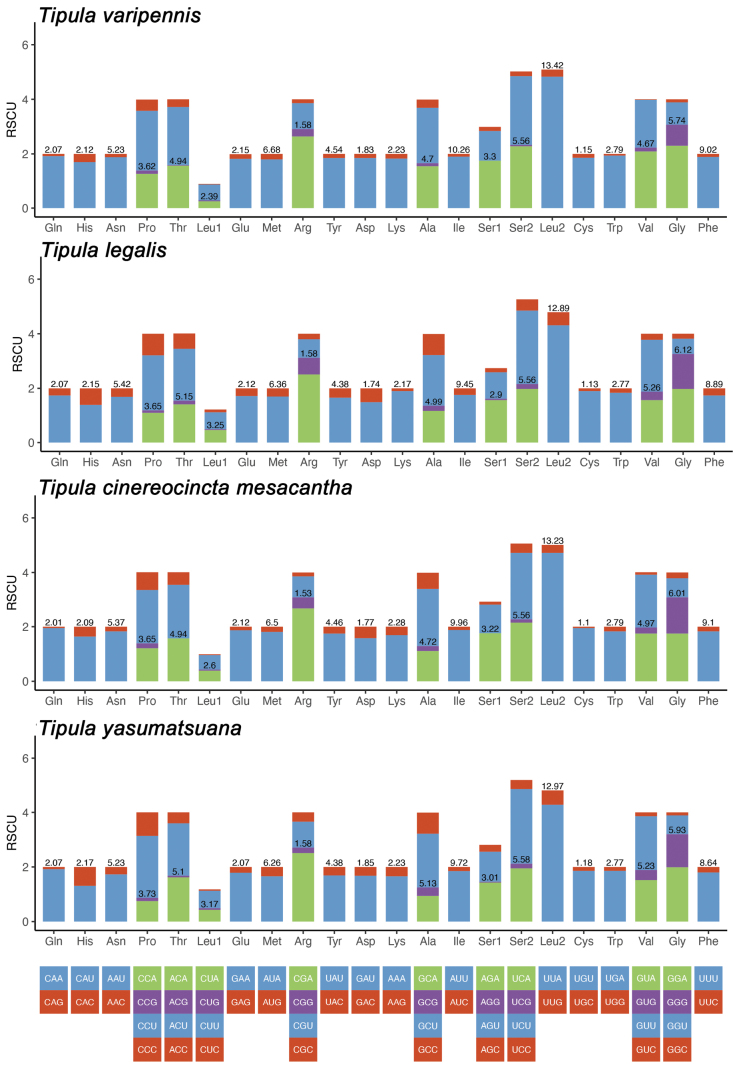
Relative synonymous codon usage (RSCU) in the mitogenomes of the four T. (Pterelachisus) species. Codes as follows: A: Ala; C: Cys; D: Asp; E: Glu; F: Phe; G: Gly; H: His; I: Ile; K: Lys; L: Leu; M: Met; N: Asn; P: Pro; Q: Gln; R: Arg; S: Ser; T: Thr; V: Val; W: Try; Y: Tyr.

All four mitochondrial genomes harbor 13 protein-coding genes, including *COX1*, *COX2*, *COX3*, *CYTB*, *ATP6*, *ATP8*, *ND2*, *ND3*, and *ND6* on the majority strand, and *ND4*, *ND4L*, *ND5*, and *ND1* on the minority strand (Fig. 1; Table 2). All species exhibit a pronounced AT richness, with A + T base content for T. (P.) cinereocincta mesacantha, T. (P.) legalis, T. (P.) varipennis, and T. (P.) yasumatsuana at 74.6%, 72.6%, 76.0%, and 73.0%, respectively. The AT richness is especially evident in third codon positions, all exceeding 80.0%, with T. (P.) varipennis having the highest value at 92.4%. The first and second codon positions have lower AT skewness values, all below 70.0%. The most frequently encoded amino acids in these four T. (Pterelachisus) mitogenomes are *Ser2*, *Leu2*, *Val*, *Gly*, *Pro*, *Thr*, *Arg*, and *Ala*, with the highest Relative Synonymous Codon Usage (RSCU) values (Fig. 2). The most common codons are UUA, AUU, UUU, and AUA, and the majority of codons are composed solely of A or T, reflecting the high AT content of protein-coding genes (PCGs).

**Figure 3.**
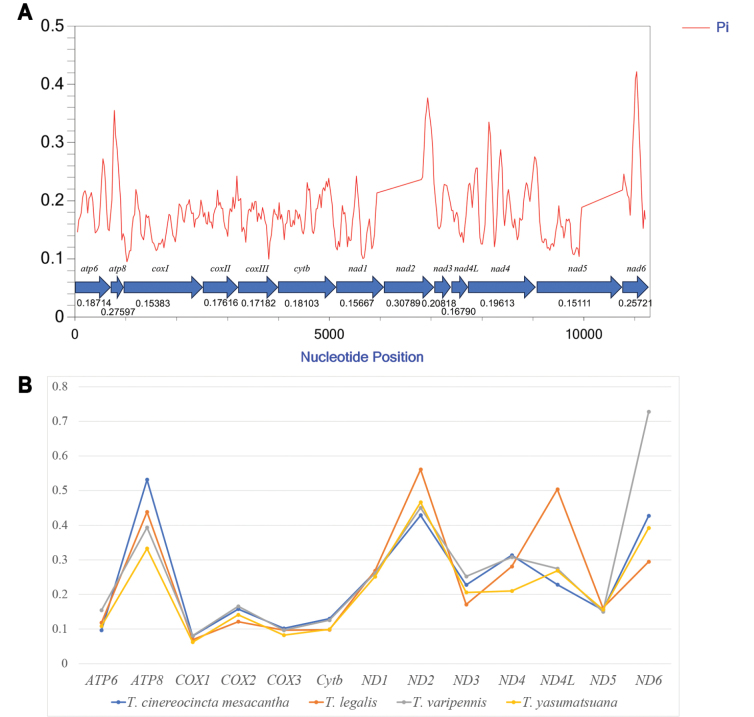
**A** The nucleotide diversity (Pi) of 13 protein-coding genes (PCGs) in four T. (Pterelachisus) species mitogenomes determined via sliding window analysis (sliding window: 100 bp; step size: 25 bp); the Pi value of each gene is shown under the gene name **B** evolutionary rates (ratios of Ka/Ks) of mitochondrial protein-coding genes of the four T. (Pterelachisus) species.

For most PCGs, typical ATN start codons (ATT / ATG) are observed in both mitochondrial genomes, except for TCG in *COX1* genes. Stop codons for most PCGs are T + tRNA, while *CYTB* has a stop codon TAG (Table 2). The sliding window analysis reveals variable nucleotide diversity (Pi) among the 13 PCGs in the four mitochondrial genomes, with *ND2* exhibiting the highest Pi (0.308), followed by *ATP8* (0.276) and *ND6* (0.257). *ND5* shows the lowest Pi (0.151) (Fig. 3A). Further examination of the Ka / Ks ratio for each PCG indicates values less than 1, suggesting purification selection. *ND2* has a notably higher Ka / Ks ratio, indicating a higher evolutionary rate, while *COX1* undergoes the highest purification selection. The Ka / Ks ratio of *ND6* varies significantly among the four species, with T. (P.) varipennis having significantly higher values than the other species (Fig. 3B, Suppl. material 2).

### ﻿Transfer RNA genes

**Figure 4.**
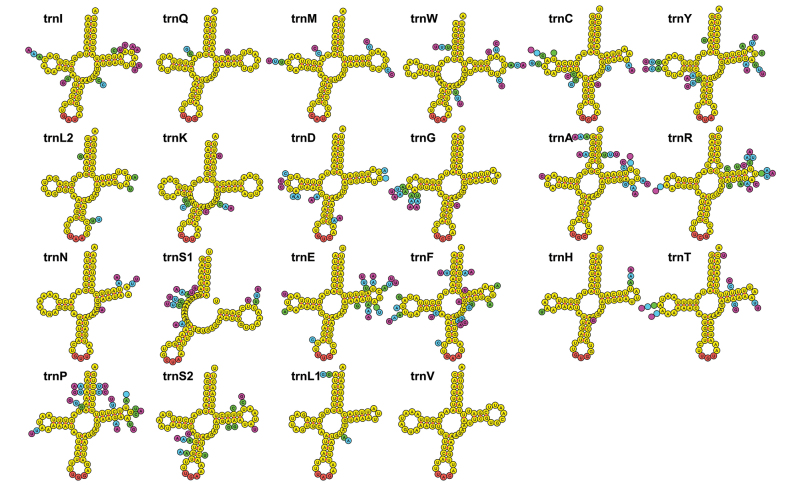
Secondary structures of tRNAs of T. (P.) varipennis. All tRNAs are labeled with the abbreviations of their corresponding amino acids. The variable sites are indicated with the green coloration for T. (P.) cinereocincta mesacantha, blue coloration for T. (P.) legalis and pink coloration for T. (P.) yasumatsuana, respectively. A blank represents a missing base site.

All mitochondrial genomes encompass 22 tRNA genes, each capable of forming cloverleaf structures, with the exception of *trnS1* (AGC), which has a dihydrouridine (DHU) arm forming a loop (Fig. 4). The length of the 22 tRNA genes ranges from 62 to 72 bp across the four mitochondrial genomes. The shortest *trnC* (GCA) genes are found in all species, with a length of 62 bp, except for *trnC* in T. (P.) varipennis, which is 63 bp. The longest tRNA genes are *trnV* (CAC) genes.

The tRNA genes in all four species exhibit significant AT richness, with A + T base content for T. (P.) cinereocincta mesacantha, T. (P.) legalis, T. (P.) varipennis, and T. (P.) yasumatsuana at 76.5%, 77.2%, 77.4%, and 76.8%, respectively (Table 3).

### ﻿Ribosomal RNA genes and non-coding regions

All four mitochondrial genomes feature two ribosomal RNA genes, *rrnL* and *rrnS*, separated by *trnV*. The *rrnL* of all four species (1,319 bp –1,323 bp) is notably longer than the *rrnS* (782 bp –783 bp). The rRNA is significantly AT-rich in all species, with A + T base content for T. (P.) cinereocincta mesacantha, T. (P.) legalis, T. (P.) varipennis, and T. (P.) yasumatsuana at 80.2%, 79.1%, 80.4%, and 79.4%, respectively (Table 3).

The control region for all four species is situated between *rrnS* and *trnI* genes, with lengths ranging from 800 bp to 1,100 bp. T. (P.) cinereocincta mesacantha has the longest control region at 1,806 bp, while T. (P.) legalis has the shortest at 805 bp. The control regions of all four species exhibit significant AT richness, with T. (P.) cinereocincta mesacantha, T. (P.) legalis, T. (P.) varipennis, and T. (P.) yasumatsuana having A + T base content of 90.7%, 91.5%, 91.4%, and 91.4%, respectively (Table 3).

**Figure 5.**
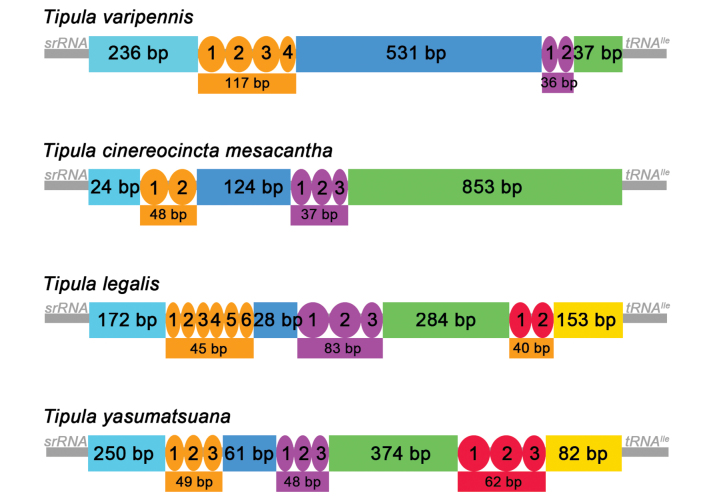
Control region structure of the four T. (Pterelachisus) species. Orange, pink, and red coloration represent tandem repeats.

The control regions for all four species were analyzed using the Tandem Repeat Finder, revealing two or three tandem repeats of varying lengths (Fig. 5). T. (P.) cinereocincta mesacantha and T. (P.) varipennis have two sets of tandem repeats, while T. (P.) legalis and T. (P.) yasumatsuana have three sets. Tandem repeats between different species exhibit no obvious common features, displaying unique structural and evolutionary characteristics.

### ﻿Phylogenetic analyses

**Figure 6.**
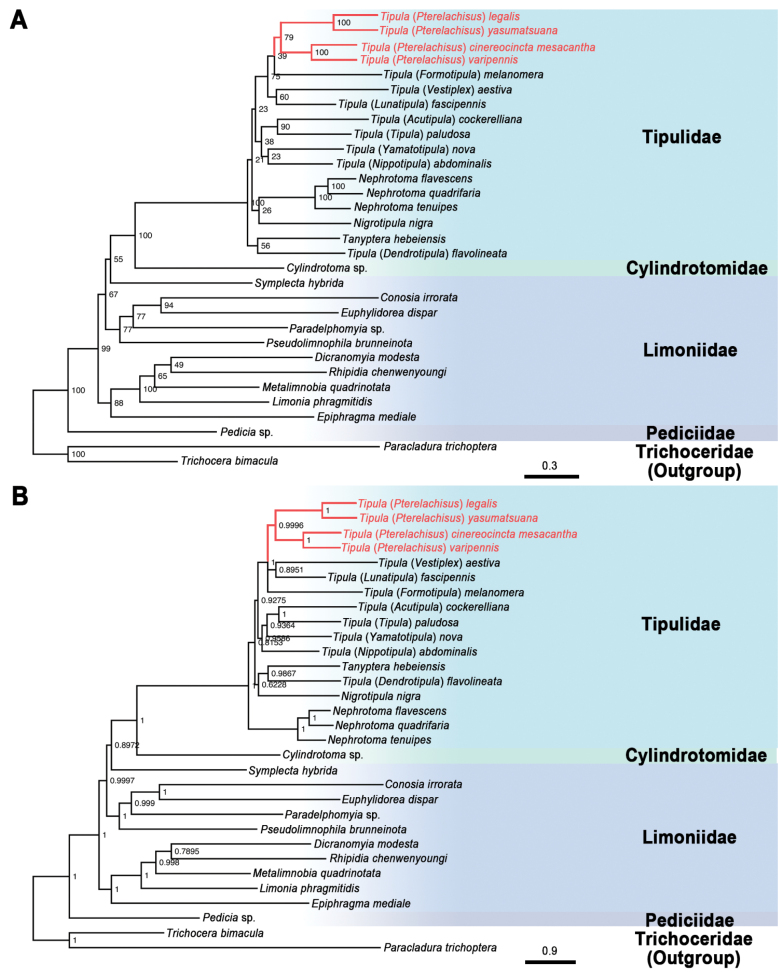
Phylogenetic trees of the selected species of Tipuloidea inferred from the datasets PCG under **A**ML and **B**BI methods. Numbers at the nodes are bootstrap values (ML tree) or posterior probabilities (BI tree). The two species of family Trichoceridae were set as the outgroups.

**Figure 7.**
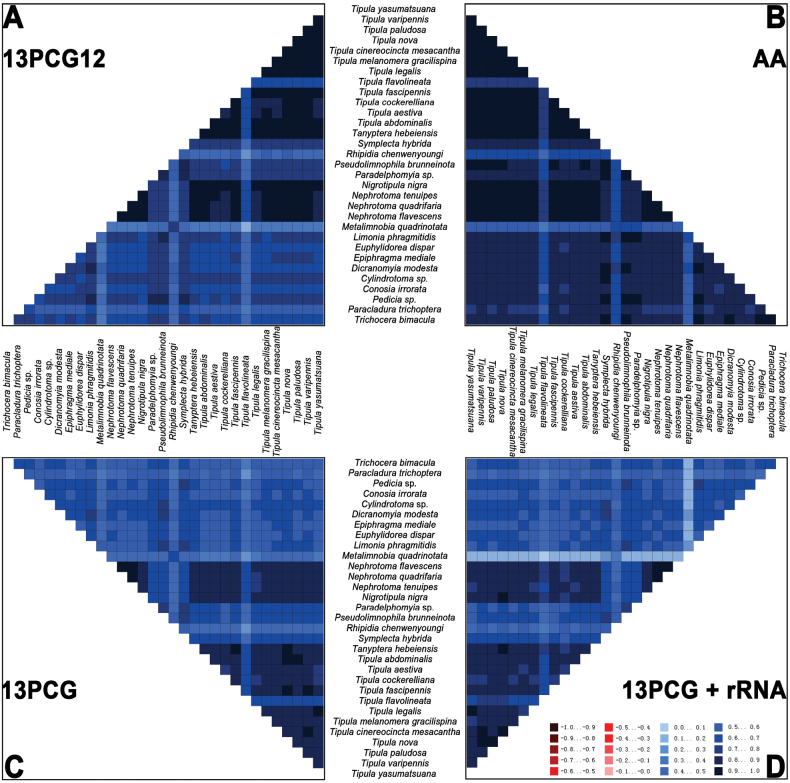
AliGROOVE analysis for four datasets. The mean similarity score between sequences is represented by a colored square, based on AliGROOVE scores ranging from -1, indicating a large difference in sequence composition from the remainder of the dataset (red coloration), to +1, indicating similarity to all other comparisons (blue coloration).

Both Bayesian inference (BI) and Maximum Likelihood (ML) trees were reconstructed using four concatenated datasets (13PCG, 13PCG12, 13PCG + rRNA, and AA) of 31 mitochondrial genomes (Fig. 6, Suppl. material 3: figs S1–S6). The topologies of these trees show notable differences. Heterogeneity in pairwise sequence differences was examined and the results have shown the AA dataset with significantly lower heterogeneity compared to the other datasets (Fig. 7), which may be a major factor influencing the phylogenetic results.

The four T. (Pterelachisus) species involved in this study are divided into two stable lineages in each phylogenetic tree above: T. (P.) cinereocincta mesacantha and T. (P.) varipennis form a sister group, while T. (P.) legalis and T. (P.) yasumatsuana form another one. Furthermore, almost all the trees, except those based on the AA dataset (Fig. 7, Suppl. material 3: figs S1, S2, S4, S5), have shown that the four T. (Pterelachisus) species compose a monophyletic lineage, but with variable support values among different trees, whereas both the ML and BI trees based on the AA dataset suggest T. (Pterelachisus) is a paraphyletic group (Suppl. material 3: figs S3, S6). Since the samples for the large subgenus T. (Pterelachisus) used in this study are far from sufficient, the monophyly of T. (Pterelachisus) needs further study with more data.

The above “*Vestiplex*-*Lunatipula*” group of the *Tipula* subgenera are tentatively supported to be a monophyletic lineage by the phylogenetic results based on the 13PCG dataset (Fig. 6), but then the subgenus T. (Formotipula), unexpectedly, should be included in this group, in addition to T. (Vestiplex), T. (Lunatipula) and T. (Pterelachisus). These arguments largely agree with the phylogenetic results based on the 13PCG + rRNA dataset (Suppl. material 3: fig. S5) in the present study, as well as the previous research results of Gao et al. (2023), only with different topologies. On the contrary, the phylogenetic trees based on the AA (Suppl. material 3: figs S3, S6) and 13PCG12 (Suppl. material 3: figs S1, S4) datasets show that the “*Vestiplex*-*Lunatipula*” group is paraphyletic, and T. (Vestiplex) is a sister-group to the remaining Tipulidae. Morphologically, T. (Pterelachisus) and other related subgenera such as T. (Vestiplex), T. (Lunatipula), and other subgenera of *Tipula* share many similarities, making it difficult to distinguish between them (Savchenko 1964; Gelhaus 1986; 2005). This supports their potential monophyly to some extent. However, it is challenging to explain the close phylogenetic relationship of T. (Formotipula) with these subgenera. Obviously, in-depth research would be required to resolve the questions on its monophyly and relationships within the group. In addition, the genus *Tipula* is not supported to be a monophyletic lineage by any of the phylogenetic trees.

The monophyly of Tipulidae and the sister relationship between Tipulidae and Cylindrotomidae are strongly supported in all BI and ML trees constructed in this study, which are consistent with the previous phylogenetic studies of Ribeiro (2008), Petersen et al. (2010), Zhang et al. (2016), and Kang et al. (2017; 2023). Tipulidae was divided into three subfamilies, i.e., Ctenophorinae, Dolichopezinae, and Tipulinae (Kertesz 1902; Oosterbroek 2024). In the present phylogenetic study, Dolichopezinae is not included due to a lack of mitogenomic data on the subfamily. Meanwhile, as the only representative of Ctenophorinae, *Tanypterahebeiensis* Yang & Yang, 1998 is sister to some members of Tipulinae (i.e., Tipula (Dendrotipula) flavolineata Meigen, 1804, Tipula (Vestiplex) aestiva Savchenko, 1960, or *Nephrotoma* spp. in different topologies) and then clustered with some other members of Tipulinae, which indicates a para- or polyphyly of the subfamily Tipulinae.

Corroborating previous phylogenetic studies (Ribeiro 2008; Petersen et al. 2010; Zhang et al. 2016; Kang et al. 2023), Limoniidae is confirmed as a non-monophyletic group in this study. Among the four traditional subfamilies of Limoniidae established by Starý (1992), three are involved in the present phylogenetic study, i.e., Chioneinae, Limnophilinae, and Limoniinae. With relatively low supporting values, *Symplectahybrida* (Meigen, 1804), the only representative of Chioneinae in this study, is sister to a clade of Cylindrotomidae + Tipulidae in almost all the trees except the BI one inferred from the dataset AA (Suppl. material 3: fig. S6). The traditional subfamily Limnophilinae is not supported as a monophyletic group, because one of its members, *Epiphragmamediale* Mao & Yang, 2019, has a relatively stable sister relationship with the clade of Limoniidae, instead of with other species of Limnophilinae, which is shown in all the BI trees and most ML trees except the one inferred from the dataset AA (Suppl. material 3: fig. S5). Similar results were also indicated in the previous studies by Kang et al. (2023) and Xu et al. (2023). Furthermore, the family Pediciidae is well supported to be sister to the remaining Tipuloidea, as all the phylogenetic analyses available on the Tipuloidea since Starý (1992).

## ﻿Conclusions

In the present study, the complete mitochondrial genomes of four T. (Pterelachisus) species were newly assembled, annotated, and characterized. Tipula (P.) varipennis was first produced as a complete circle molecular structure based on previously published raw data (MT410829, 13,483bp), while another three were sequenced and reported upon for the first time. These four mitochondrial genomes show similarities in gene order, nucleotide composition, and codon usage with those of other known crane fly species. The phylogenetic results have reconfirmed the monophyly of the family Tipulidae, the sister relationship between Tipulidae and Cylindrotomidae, and the phylogenetic status of Pediciidae as sister group to the remaining Tipuloidea. On the other hand, the monophyly of the tipulid subfamily Tipulinae or the genus *Tipula*, as well as that of Limoniidae, have not been supported, while the limoniid subfamily Limnophilinae has been suggested as a polyphyletic group. The subgenus T. (Pterelachisus) might be a monophyletic lineage according to current mitogenome data, whereas it is not stable enough. Moreover, it has shown closer phylogenetic relationships between T. (Pterelachisus) and the subgenera T. (Formotipula), T. (Lunatipula), and T. (Vestiplex). The phylogenetic status of T. (Pterelachisus) in Tipulidae is under analysis using different mitogenomic datasets: both the ML and BI trees inferred from the AA dataset have shown more divergent topologies from other trees, probably due to the relatively lower heterogeneity of the dataset.

It is evident that the tiny number of samples is insufficient for a thorough phylogenetic analysis of the vast crane fly group. It is noteworthy to remember that, particularly in cases where the sample number is limited and replicates are few, mitochondrial genotyping may not be entirely successful in resolving deep phylogenetic relationships. This could lead to low support for particular evolutionary branches, which would impair the precision of the findings. However, this study provides new insights into the phylogenetic relationships within Tipulidae, particularly on T. (Pterelachisus). To better understand the phylogeny of crane flies, more samples covering a broader range of taxa will be necessary in the future study.

## ﻿Additional information

### Author ORCIDs

Yuetian Gao https://orcid.org/0000-0003-2966-1745

Wanxin Cai https://orcid.org/0000-0001-6604-4899

Yupeng Li https://orcid.org/0009-0008-5381-2260

Yan Li https://orcid.org/0000-0002-4896-5843

Ding Yang https://orcid.org/0000-0002-7685-3478

### Data availability

All of the data that support the findings of this study are available in the main text or Supplementary Information.

## Supplementary materials

Supplementary material 1 Information of the voucher specimens used for mitochondrial genomes sequencing in the present study Data type docx https://binary.pensoft.net/file/1143147This dataset is made available under the Open Database License (http://opendatacommons.org/licenses/odbl/1.0/). The Open Database License (ODbL) is a license agreement intended to allow users to freely share, modify, and use this Dataset while maintaining this same freedom for others, provided that the original source and author(s) are credited.Yuetian Gao, Wanxin Cai, Yupeng Li, Yan Li, Ding Yang

Supplementary material 2 Synonymous and non-synonymous substitutional analysis of gene *ATP6*, *ATP8*, *COX1*, *COX2*, *COX3*, *CYTB*, *ND1*, *ND2*, *ND3*, *ND4*, *ND4L*, *ND5*, *ND6* Data type docx https://binary.pensoft.net/file/1143148This dataset is made available under the Open Database License (http://opendatacommons.org/licenses/odbl/1.0/). The Open Database License (ODbL) is a license agreement intended to allow users to freely share, modify, and use this Dataset while maintaining this same freedom for others, provided that the original source and author(s) are credited.Yuetian Gao, Wanxin Cai, Yupeng Li, Yan Li, Ding Yang

Supplementary material 3 Phylogenetic trees Data type pdf Explanation note **figure S1.** Phylogenetic trees of the selected species of Tipuloidea inferred from the datasets 13PCG12 under ML methods. Numbers at the nodes are bootstrap values. The two species of family Trichoceridae were set as the outgroups; **figure S2.** Phylogenetic trees of the selected species of Tipuloidea inferred from the datasets 13PCG+rRNA under ML methods. Numbers at the nodes are bootstrap values. The two species of family Trichoceridae were set as the outgroup; **figure S3.** Phylogenetic trees of the selected species of Tipuloidea inferred from the datasets AA under ML methods. Numbers at the nodes are bootstrap values. The two species of family Trichoceridae were set as the outgroups; **figure S4.** Phylogenetic trees of the selected species of Tipuloidea inferred from the datasets 13PCG12 under BI methods. Numbers at the nodes are posterior probabilities. The two species of family Trichoceridae were set as the outgroups; **figure S5.** Phylogenetic trees of the selected species of Tipuloidea inferred from the datasets 13PCG+rRNA under BI methods. Numbers at the nodes are posterior probabilities. The two species of family Trichoceridae were set as the outgroups; **figure S6.** Phylogenetic trees of the selected species of Tipuloidea inferred from the datasets AA under BI methods. Numbers at the nodes are posterior probabilities. The two species of family Trichoceridae were set as the outgroups. https://binary.pensoft.net/file/1143149This dataset is made available under the Open Database License (http://opendatacommons.org/licenses/odbl/1.0/). The Open Database License (ODbL) is a license agreement intended to allow users to freely share, modify, and use this Dataset while maintaining this same freedom for others, provided that the original source and author(s) are credited.Yuetian Gao, Wanxin Cai, Yupeng Li, Yan Li, Ding Yang
