# Supplementary material for: ﻿Four complete mitochondrial genomes of the subgenus Pterelachisus (Diptera, Tipulidae, Tipula) and implications for the higher phylogeny of the family Tipulidae
Source: Zookeys. 2024 Sep 27;1213:267–88. doi: 10.3897/zookeys.1213.122708 (PMC11452739; doi:10.3897/zookeys.1213.122708)
Supplement: Supplementary material 1 — Information of the voucher specimens used for mitochondrial genomes sequencing in the present study [file zookeys-1213-267_article-122708__-s001.docx]

**Table S1.** Information of the voucher specimens used for mitochondrial genomes sequencing in the present study.

| Species | Location | Collection Date |
| --- | --- | --- |
| *T.* (*P.*) *cinereocincta mesacantha* | CHINA, Liaoning, Fushun | 2021.VII.23 |
| *T.* (*P*.) *legalis* | CHINA, Hebei, Shijiazhuang | 2016.VII.8 |
| *T.* (*P*.) *yasumatsuana* | CHINA, Hebei, Xinglongxian | 2018.VI.1 |
