# Supplementary material for: ﻿Four complete mitochondrial genomes of the subgenus Pterelachisus (Diptera, Tipulidae, Tipula) and implications for the higher phylogeny of the family Tipulidae
Source: Zookeys. 2024 Sep 27;1213:267–88. doi: 10.3897/zookeys.1213.122708 (PMC11452739; doi:10.3897/zookeys.1213.122708)
Supplement: Supplementary material 2 — Synonymous and non-synonymous substitutional analysis of gene ATP6, ATP8, COX1, COX2, COX3, CYTB, ND1, ND2, ND3, ND4, ND4L, ND5, ND6 [file zookeys-1213-267_article-122708__-s002.docx]

**Table** **S2-1.** Synonymous and nonsynonymous substitutional analysis of gene ATP6.

| Species | SynDif | SynPos | Ks | NSynDif | NSynPos | Ka | Ka/Ks |
| --- | --- | --- | --- | --- | --- | --- | --- |
| *T. cinereocincta mesacantha* | 92.83 | 158 | 1.1473 | 53.17 | 517 | 0.1106 | 0.09640024 |
| *T. legalis* | 87.83 | 158.17 | 1.0115 | 57.17 | 516.83 | 0.1197 | 0.1183391 |
| *T. varipennis* | 75.50 | 157.5 | 0.7645 | 56.50 | 517.5 | 0.118 | 0.15434925 |
| *T. yasumatsuana* | 86.83 | 157.5 | 0.9963 | 52.17 | 517.5 | 0.1083 | 0.1087022 |

Abbreviations: SynDif, the total number of synonymous differences; SynPos, the total number of synonymous sites; Ks, the number of synonymous (or silent) substitutions per synonymous (or silent) site; NSynDif, the total number of nonsynonymous differences; NSynPos, the total number of nonsynonymous sites; Ka, the number of nonsynonymous substitutions per nonsynonymous site.

**Table S2-2.** Synonymous and nonsynonymous substitutional analysis of gene ATP8.

| Species | SynDif | SynPos | Ks | NSynDif | NSynPos | Ka | Ka/Ks |
| --- | --- | --- | --- | --- | --- | --- | --- |
| *T. cinereocincta mesacantha* | 13.50 | 34.33 | 0.5572 | 30.50 | 124.67 | 0.2961 | 0.53140704 |
| *T. legalis* | 15.08 | 33.67 | 0.6823 | 30.92 | 125.33 | 0.2991 | 0.43837022 |
| *T. varipennis* | 16.83 | 34.17 | 0.8023 | 32.17 | 124.83 | 0.3157 | 0.39349371 |
| *T. yasumatsuana* | 20.00 | 35.67 | 1.0327 | 34.00 | 123.33 | 0.3436 | 0.33272005 |

Abbreviations: SynDif, the total number of synonymous differences; SynPos, the total number of synonymous sites; Ks, the number of synonymous (or silent) substitutions per synonymous (or silent) site; NSynDif, the total number of nonsynonymous differences; NSynPos, the total number of nonsynonymous sites; Ka, the number of nonsynonymous substitutions per nonsynonymous site.

**Table S2-3.** Synonymous and nonsynonymous substitutional analysis of gene COX1.

| Species | SynDif | SynPos | Ks | NSynDif | NSynPos | Ka | Ka/Ks |
| --- | --- | --- | --- | --- | --- | --- | --- |
| *T. cinereocincta mesacantha* | 166.83 | 353.83 | 0.743 | 65.17 | 1143.17 | 0.0593 | 0.07981157 |
| *T. legalis* | 179.83 | 353 | 0.8528 | 65.17 | 1144 | 0.0592 | 0.06941839 |
| *T. varipennis* | 167.83 | 352.67 | 0.7549 | 67.17 | 1144.33 | 0.0611 | 0.08093787 |
| *T. yasumatsuana* | 183.33 | 353.17 | 0.8836 | 60.67 | 1143.83 | 0.055 | 0.06224536 |

Abbreviations: SynDif, the total number of synonymous differences; SynPos, the total number of synonymous sites; Ks, the number of synonymous (or silent) substitutions per synonymous (or silent) site; NSynDif, the total number of nonsynonymous differences; NSynPos, the total number of nonsynonymous sites; Ka, the number of nonsynonymous substitutions per nonsynonymous site.

**Table S2-4.** Synonymous and nonsynonymous substitutional analysis of gene COX2.

| Species | SynDif | SynPos | Ks | NSynDif | NSynPos | Ka | Ka/Ks |
| --- | --- | --- | --- | --- | --- | --- | --- |
| *T. cinereocincta mesacantha* | 67.00 | 144.67 | 0.7208 | 56.00 | 530.33 | 0.1138 | 0.15788013 |
| *T. legalis* | 79.00 | 146 | 0.9586 | 57.00 | 529 | 0.1163 | 0.12132276 |
| *T. varipennis* | 64.00 | 144 | 0.6735 | 55.00 | 531 | 0.1115 | 0.16555308 |
| *T. yasumatsuana* | 72.83 | 145.5 | 0.8257 | 57.17 | 529.5 | 0.1166 | 0.14121352 |

Abbreviations: SynDif, the total number of synonymous differences; SynPos, the total number of synonymous sites; Ks, the number of synonymous (or silent) substitutions per synonymous (or silent) site; NSynDif, the total number of nonsynonymous differences; NSynPos, the total number of nonsynonymous sites; Ka, the number of nonsynonymous substitutions per nonsynonymous site.

**Table S2-5.** Synonymous and nonsynonymous substitutional analysis of gene COX3.

| Species | SynDif | SynPos | Ks | NSynDif | NSynPos | Ka | Ka/Ks |
| --- | --- | --- | --- | --- | --- | --- | --- |
| *T. cinereocincta mesacantha* | 87.83 | 177.67 | 0.8073 | 47.17 | 605.33 | 0.0823 | 0.10194475 |
| *T. legalis* | 92.50 | 179 | 0.876 | 48.50 | 604 | 0.0849 | 0.09691781 |
| *T. varipennis* | 83.00 | 175.17 | 0.7493 | 42.00 | 607.83 | 0.0725 | 0.09675697 |
| *T. yasumatsuana* | 99.67 | 176.83 | 1.0442 | 49.33 | 606.17 | 0.0861 | 0.08245547 |

Abbreviations: SynDif, the total number of synonymous differences; SynPos, the total number of synonymous sites; Ks, the number of synonymous (or silent) substitutions per synonymous (or silent) site; NSynDif, the total number of nonsynonymous differences; NSynPos, the total number of nonsynonymous sites; Ka, the number of nonsynonymous substitutions per nonsynonymous site.

**Table S2-6.** Synonymous and nonsynonymous substitutional analysis of gene CYTB.

| Species | SynDif | SynPos | Ks | NSynDif | NSynPos | Ka | Ka/Ks |
| --- | --- | --- | --- | --- | --- | --- | --- |
| *T. cinereocincta mesacantha* | 123 | 254 | 0.7781 | 83.00 | 877 | 0.1012 | 0.1300604 |
| *T. legalis* | 137.17 | 254.5 | 0.951 | 76.83 | 876.5 | 0.0932 | 0.0980021 |
| *T. varipennis* | 122.5 | 252.83 | 0.7789 | 80.50 | 878.17 | 0.0978 | 0.12556169 |
| *T. yasumatsuana* | 137.83 | 256.5 | 0.9454 | 77.17 | 874.5 | 0.0939 | 0.09932304 |

Abbreviations: SynDif, the total number of synonymous differences; SynPos, the total number of synonymous sites; Ks, the number of synonymous (or silent) substitutions per synonymous (or silent) site; NSynDif, the total number of nonsynonymous differences; NSynPos, the total number of nonsynonymous sites; Ka, the number of nonsynonymous substitutions per nonsynonymous site.

**Table S2-7.** Synonymous and nonsynonymous substitutional analysis of gene ND1.

| Species | SynDif | SynPos | Ks | NSynDif | NSynPos | Ka | Ka/Ks |
| --- | --- | --- | --- | --- | --- | --- | --- |
| *T. cinereocincta mesacantha* | 70.42 | 206.5 | 0.4548 | 79.58 | 717.5 | 0.12 | 0.26385224 |
| *T. legalis* | 73.92 | 206.33 | 0.4871 | 86.08 | 717.67 | 0.1307 | 0.26832273 |
| *T. varipennis* | 71.58 | 205 | 0.4699 | 81.42 | 719 | 0.1228 | 0.2613322 |
| *T. yasumatsuana* | 74.08 | 207 | 0.4864 | 80.92 | 717 | 0.1223 | 0.25143914 |

Abbreviations: SynDif, the total number of synonymous differences; SynPos, the total number of synonymous sites; Ks, the number of synonymous (or silent) substitutions per synonymous (or silent) site; NSynDif, the total number of nonsynonymous differences; NSynPos, the total number of nonsynonymous sites; Ka, the number of nonsynonymous substitutions per nonsynonymous site.

**Table S2-8.** Synonymous and nonsynonymous substitutional analysis of gene ND2.

| Species | SynDif | SynPos | Ks | NSynDif | NSynPos | Ka | Ka/Ks |
| --- | --- | --- | --- | --- | --- | --- | --- |
| *T. cinereocincta mesacantha* | 19.17 | 38.67 | 0.8111 | 41.83 | 150.33 | 0.3478 | 0.42880039 |
| *T. legalis* | 17.17 | 40.33 | 0.6286 | 41.83 | 148.67 | 0.3527 | 0.56108813 |
| *T. varipennis* | 18.17 | 39.00 | 0.7278 | 39.83 | 150 | 0.3278 | 0.45039846 |
| *T. yasumatsuana* | 19.17 | 39.67 | 0.7752 | 42.83 | 149.33 | 0.3615 | 0.46633127 |

Abbreviations: SynDif, the total number of synonymous differences; SynPos, the total number of synonymous sites; Ks, the number of synonymous (or silent) substitutions per synonymous (or silent) site; NSynDif, the total number of nonsynonymous differences; NSynPos, the total number of nonsynonymous sites; Ka, the number of nonsynonymous substitutions per nonsynonymous site.

**Table S2-9.** Synonymous and nonsynonymous substitutional analysis of gene ND3.

| Species | SynDif | SynPos | Ks | NSynDif | NSynPos | Ka | Ka/Ks |
| --- | --- | --- | --- | --- | --- | --- | --- |
| *T. cinereocincta mesacantha* | 34.17 | 70.17 | 0.7858 | 41.83 | 262.83 | 0.1789 | 0.22766607 |
| *T. legalis* | 38.83 | 70.33 | 0.9994 | 40.17 | 262.67 | 0.171 | 0.17110266 |
| *T. varipennis* | 33.33 | 70.50 | 0.7465 | 43.67 | 262.5 | 0.1881 | 0.25197589 |
| *T. yasumatsuana* | 38.33 | 71.50 | 0.941 | 44.67 | 261.5 | 0.1938 | 0.20595112 |

Abbreviations: SynDif, the total number of synonymous differences; SynPos, the total number of synonymous sites; Ks, the number of synonymous (or silent) substitutions per synonymous (or silent) site; NSynDif, the total number of nonsynonymous differences; NSynPos, the total number of nonsynonymous sites; Ka, the number of nonsynonymous substitutions per nonsynonymous site.

**Table S2-10.** Synonymous and nonsynonymous substitutional analysis of gene ND4.

| Species | SynDif | SynPos | Ks | NSynDif | NSynPos | Ka | Ka/Ks |
| --- | --- | --- | --- | --- | --- | --- | --- |
| *T. cinereocincta mesacantha* | 110.33 | 292.67 | 0.5239 | 153.67 | 1042.33 | 0.1641 | 0.31322772 |
| *T. legalis* | 122.33 | 295.17 | 0.6032 | 157.67 | 1039.83 | 0.1694 | 0.28083554 |
| *T. varipennis* | 111.5 | 292.5 | 0.5324 | 153.5 | 1042.5 | 0.1639 | 0.30785124 |
| *T. yasumatsuana* | 143.67 | 295.83 | 0.7821 | 153.33 | 1039.17 | 0.1643 | 0.21007544 |

Abbreviations: SynDif, the total number of synonymous differences; SynPos, the total number of synonymous sites; Ks, the number of synonymous (or silent) substitutions per synonymous (or silent) site; NSynDif, the total number of nonsynonymous differences; NSynPos, the total number of nonsynonymous sites; Ka, the number of nonsynonymous substitutions per nonsynonymous site.

**Table S2-11.** Synonymous and nonsynonymous substitutional analysis of gene ND4L.

| Species | SynDif | SynPos | Ks | NSynDif | NSynPos | Ka | Ka/Ks |
| --- | --- | --- | --- | --- | --- | --- | --- |
| *T. cinereocincta mesacantha* | 19.67 | 52.33 | 0.5215 | 21.33 | 193.67 | 0.1191 | 0.22837967 |
| *T. legalis* | 12.67 | 53.17 | 0.2867 | 25.33 | 192.83 | 0.1444 | 0.50366236 |
| *T. varipennis* | 15.67 | 51.67 | 0.3885 | 19.33 | 194.33 | 0.1067 | 0.27464607 |
| *T. yasumatsuana* | 17.67 | 52.67 | 0.4446 | 21.33 | 193.33 | 0.1194 | 0.26855601 |

Abbreviations: SynDif, the total number of synonymous differences; SynPos, the total number of synonymous sites; Ks, the number of synonymous (or silent) substitutions per synonymous (or silent) site; NSynDif, the total number of nonsynonymous differences; NSynPos, the total number of nonsynonymous sites; Ka, the number of nonsynonymous substitutions per nonsynonymous site.

**Table S2-12.** Synonymous and nonsynonymous substitutional analysis of gene ND5.

| Species | SynDif | SynPos | Ks | NSynDif | NSynPos | Ka | Ka/Ks |
| --- | --- | --- | --- | --- | --- | --- | --- |
| *T. cinereocincta mesacantha* | 84.83 | 204.67 | 0.6033 | 65.17 | 740.33 | 0.0936 | 0.15514669 |
| *T. legalis* | 87.00 | 206.67 | 0.6179 | 69.00 | 738.33 | 0.0998 | 0.16151481 |
| *T. varipennis* | 82.33 | 206.17 | 0.5702 | 59.67 | 738.83 | 0.0854 | 0.14977201 |
| *T. yasumatsuana* | 87.33 | 206 | 0.6248 | 67.67 | 739 | 0.0977 | 0.15637004 |

Abbreviations: SynDif, the total number of synonymous differences; SynPos, the total number of synonymous sites; Ks, the number of synonymous (or silent) substitutions per synonymous (or silent) site; NSynDif, the total number of nonsynonymous differences; NSynPos, the total number of nonsynonymous sites; Ka, the number of nonsynonymous substitutions per nonsynonymous site.

**Table S2-13.** Synonymous and nonsynonymous substitutional analysis of gene ND6.

| Species | SynDif | SynPos | Ks | NSynDif | NSynPos | Ka | Ka/Ks |
| --- | --- | --- | --- | --- | --- | --- | --- |
| *T. cinereocincta mesacantha* | 46.67 | 100 | 0.7301 | 102.33 | 401 | 0.3119 | 0.42720175 |
| *T. legalis* | 58.67 | 102.5 | 1.0802 | 103.33 | 398.5 | 0.3182 | 0.29457508 |
| *T. varipennis* | 32.50 | 99.50 | 0.4289 | 102.5 | 401.5 | 0.3121 | 0.72767545 |
| *T. yasumatsuana* | 49.33 | 101.5 | 0.7832 | 100.67 | 399.5 | 0.3071 | 0.3921093 |

Abbreviations: SynDif, the total number of synonymous differences; SynPos, the total number of synonymous sites; Ks, the number of synonymous (or silent) substitutions per synonymous (or silent) site; NSynDif, the total number of nonsynonymous differences; NSynPos, the total number of nonsynonymous sites; Ka, the number of nonsynonymous substitutions per nonsynonymous site.
