# Supplementary figures and images for: ﻿Four complete mitochondrial genomes of the subgenus Pterelachisus (Diptera, Tipulidae, Tipula) and implications for the higher phylogeny of the family Tipulidae
Source: Zookeys. 2024 Sep 27;1213:267–88. doi: 10.3897/zookeys.1213.122708 (PMC11452739; doi:10.3897/zookeys.1213.122708)

SF1

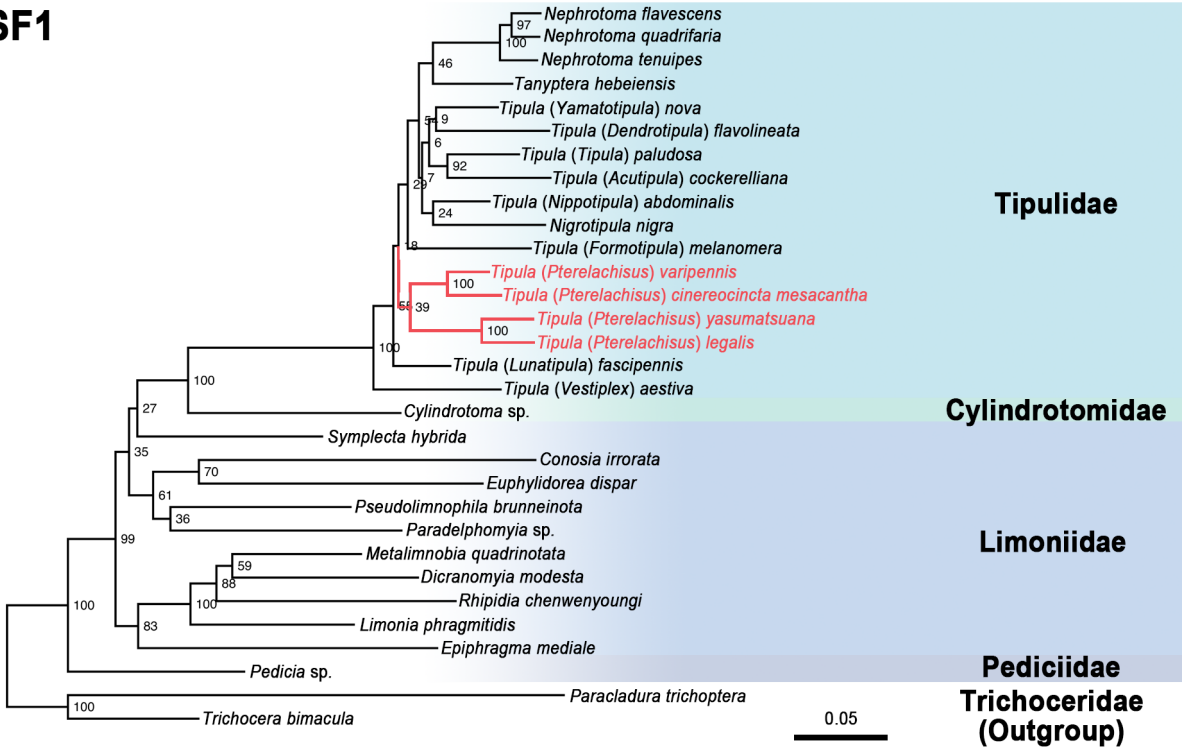

SF2

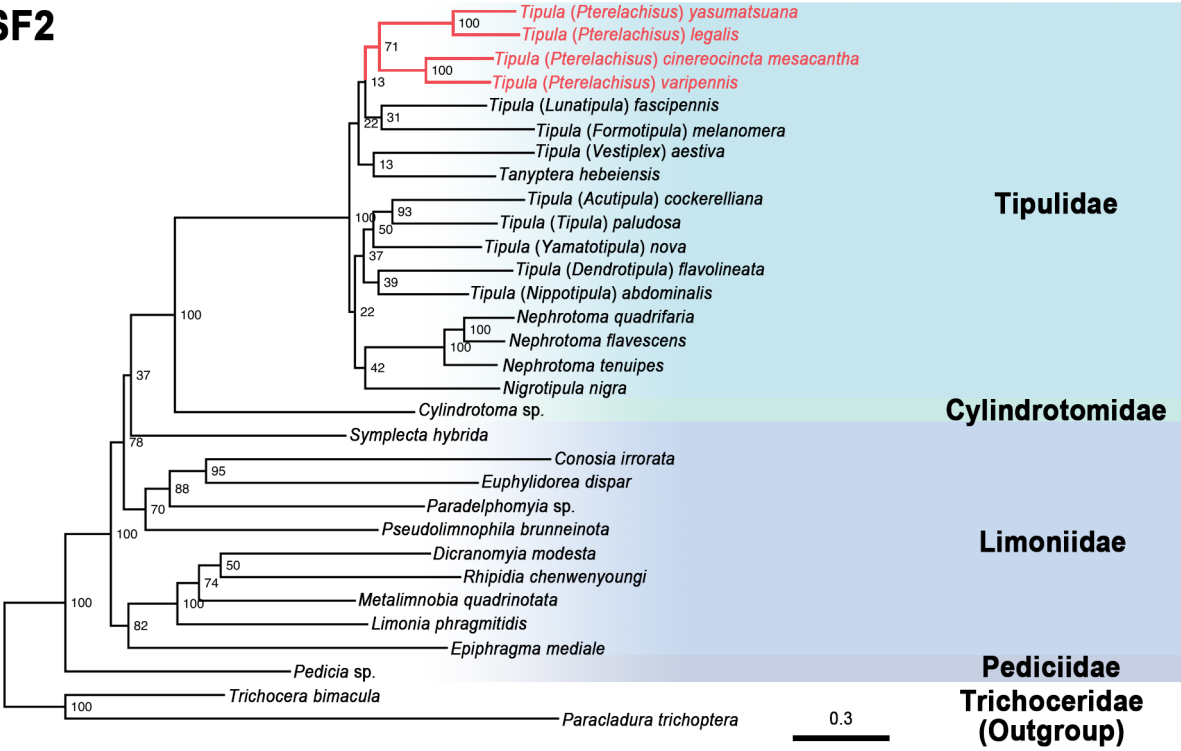

SF3

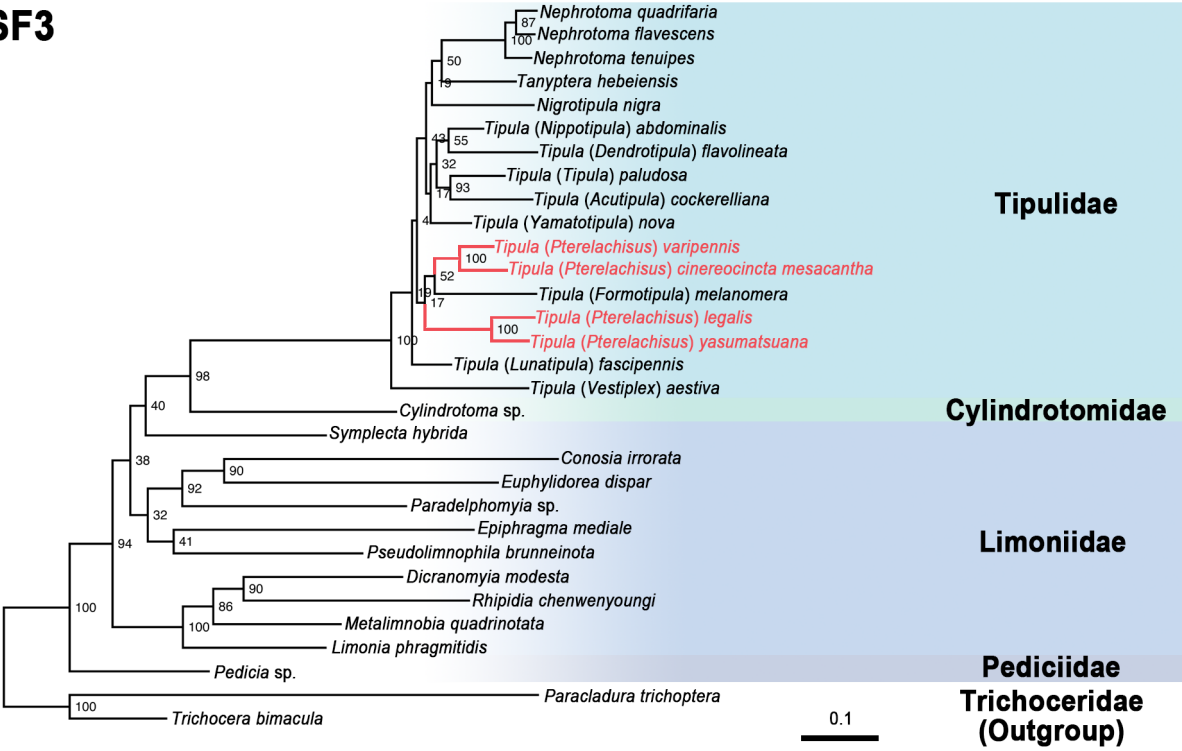

SF4

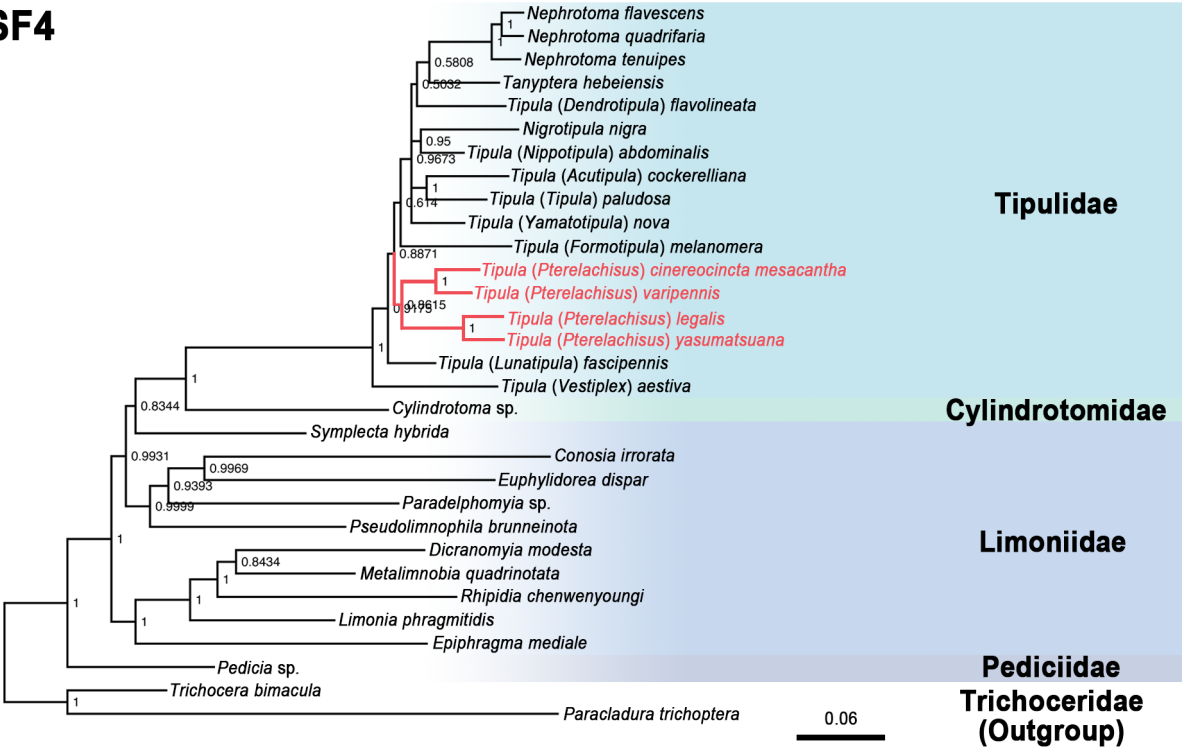

SF5

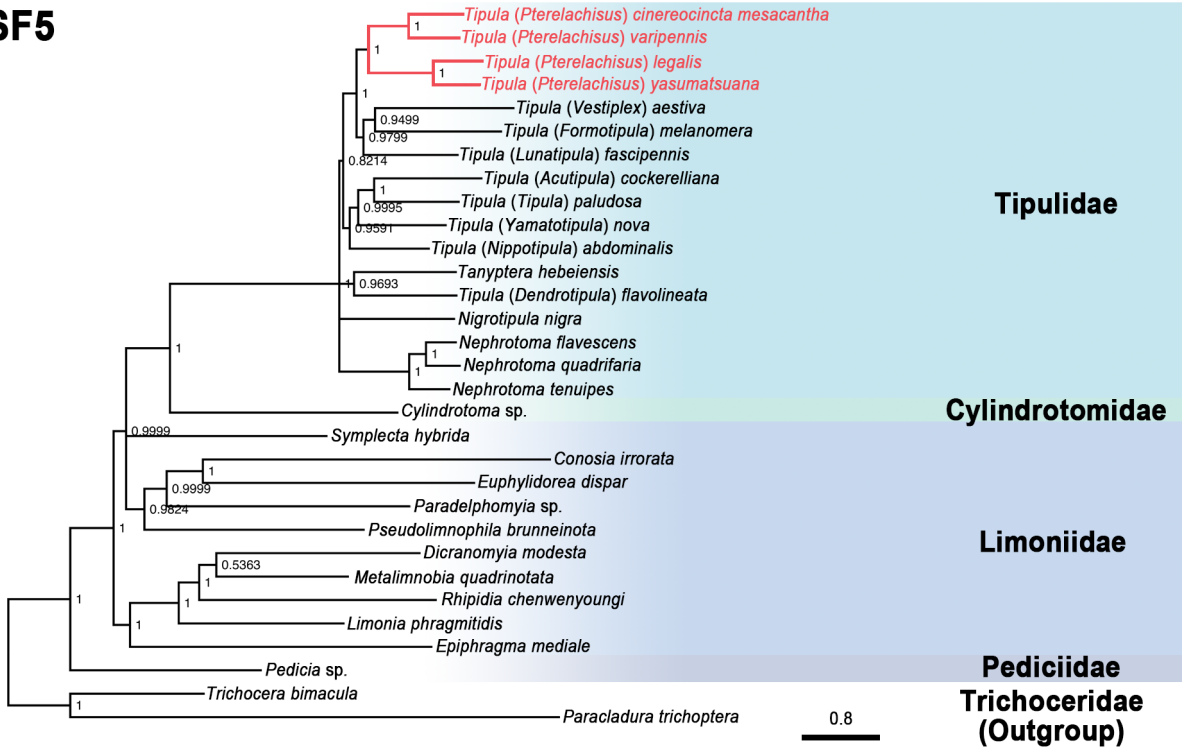

SF6

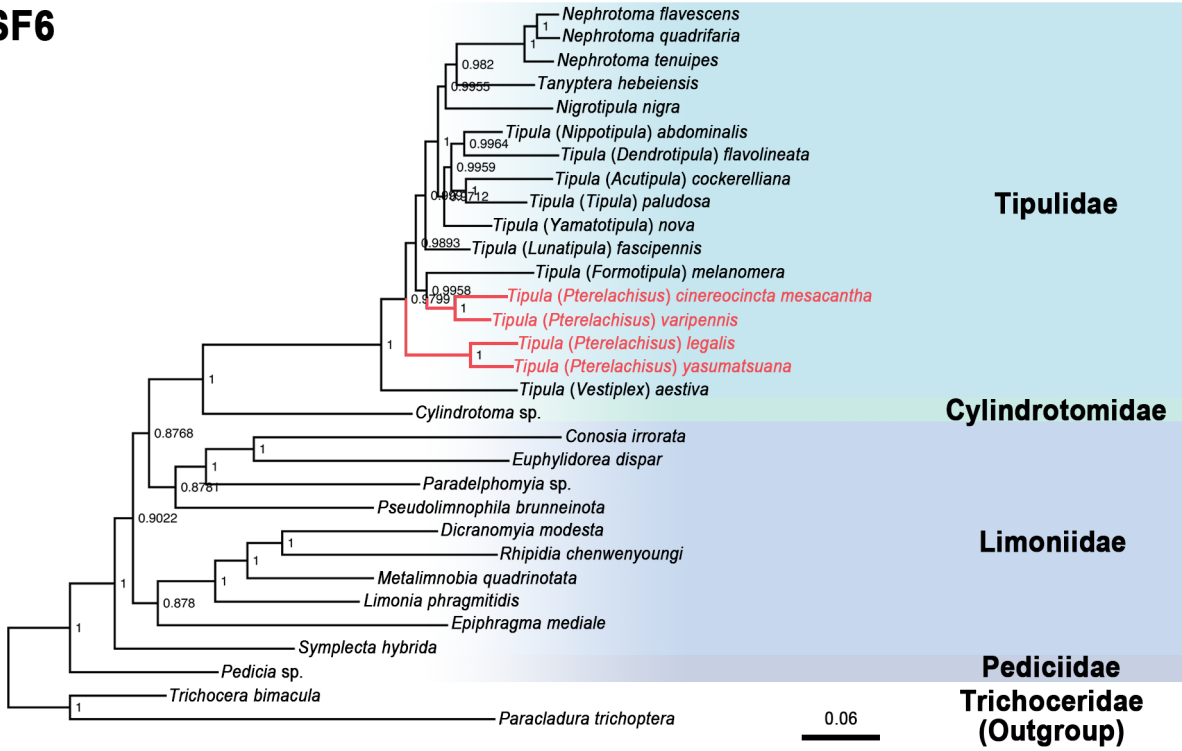

Supplement: Supplementary material 3 — Phylogenetic trees [file zookeys-1213-267_article-122708__-s003.pdf]
